# Professional benefits perception among pediatric nurses: a cross-sectional survey

**DOI:** 10.3389/fped.2025.1539376

**Published:** 2025-03-05

**Authors:** Jia Wang, Hui Wang, Xiuli Chen, Yu Sheng

**Affiliations:** Department of General Surgery, Children’s Hospital of Nanjing Medical University, Nanjing, Jiangsu, China

**Keywords:** professional benefit perception, pediatric, nurse, care, nursing, management

## Abstract

**Background:**

The perception of professional benefits is a critical determinant of job stability and work efficacy among healthcare professionals. This perception can directly influence patient care quality, nurse job satisfaction and retention, as well as overall organizational efficiency. The current understanding of how pediatric nurses perceive their professional benefits is limited. This study aimed to elucidate the status and determinants of professional benefit perception among pediatric nurses to inform evidence-based clinical care.

**Methods:**

We conducted a survey of pediatric nurses at our hospital from September 1, 2024, to November 26, 2024. Participants were selected using a convenience sampling method. The Nurses' Professional benefit perception Scale was employed to assess professional benefit perception. Pearson correlation and multiple linear regression analyses were utilized to identify factors influencing professional benefit perception. These statistical methods were chosen due to their suitability for examining relationships between continuous variables and for controlling potential confounding factors in the regression model.

**Results:**

A total of 366 pediatric nurses participated in the study. The mean score of professional benefit perception was (108.26 ± 17.32). Pearson correlation analysis revealed significant positive associations between professional benefit perception scores and several demographic and professional variables. Specifically, age (*r* = 0.517), years of experience in pediatric nursing (*r* = 0.604), educational level (*r* = 0.564), professional title (*r* = 0.559), marital status (*r* = 0.623), and average monthly income (*r* = 0.647) were all significantly correlated with professional benefit perception scores. Further analysis using multiple linear regression identified these variables as significant predictors of professional benefit perception scores, collectively accounting for 57.20% of the variance (*R*² = 0.572). This indicates that a substantial portion of the variation in professional benefit perception can be explained by these demographic and professional factors.

**Conclusion:**

The perceived level of professional benefits among pediatric nurses is found to be moderate. It is recommended that nursing administrators develop targeted intervention strategies based on the identified influencing factors. Such strategies may include enhancing professional development opportunities, improving financial compensation, and fostering a supportive work environment.

## Background

With the full implementation of the two-child policy in China, there has been a rapid increase in the demand for children's medical and health services (1). Following the policy change, there has been a notable increase in birth rates, particularly in multiple births. This has led to a growing demand for pediatric healthcare services, placing additional pressure on the existing pediatric nursing workforce. For example, data from a nationwide interrupted time-series analysis shows that the total number of births, including preterm and multiple births, has risen significantly since the policy was introduced (2). This trend underscores the urgency of addressing the challenges faced by pediatric nursing in China. Globally, the distribution of pediatric healthcare professionals is highly uneven, with low-income countries often experiencing significant shortages. For instance, in some regions, the number of pediatricians per 100,000 children is as low as 0.5, compared to 72 in high-income countries (3). This disparity highlights the unique challenges faced by China, where the rapid increase in pediatric healthcare demand due to the two-child policy further exacerbates existing workforce shortages (4).

In their daily practice, pediatric nurses are confronted with multifaceted challenges. These include managing uncooperative young patients, navigating a noisy work environment, coping with heavy workloads, and addressing complex nurse-patient relationships (5, 6). Moreover, the demanding nature of their work is not fully matched by their remuneration, leading to a gradual decline in the enthusiasm and proactivity of many pediatric nurses (7). Studies (8–10) indicate that the rate of job burnout among pediatric nurses is as high as 76.25%, with 14.02% experiencing severe burnout. Recognizing that work stress is an inevitable part of the profession, there is an urgent need to address the long-standing psychological issues faced by nursing teams from a positive perspective, in order to promote the physical and mental well-being of nurses and the stability of the team.

The professional benefit perception in nursing refers to the perceived rewards and benefits that nurses experience throughout their careers, as well as their belief that engaging in nursing work can promote their overall personal development (11, 12). This sense of gain is a form of occupational sentiment shaped under the influence of positive psychology, serving as an intrinsic motivational mechanism that positively affects nurses' professional attitudes and behaviors (13). When nurses assess the value of their labor and the supportive nature of their work environment, and perceive that their needs at various levels are met, they experience a sense of spiritual welfare. Research (14) indicates that the experience of occupational emotions can significantly alter practitioners' attitudes towards their work, with the tangible benefits of the profession capable of evoking positive emotions and potentially transforming initial resistance into affection.

Although existing studies (15, 16) have explored the advantages and sense of gain in the nursing profession, they have primarily focused on specific areas such as clinical teaching nurses and palliative care nurses. These studies provide valuable insights but fail to comprehensively address the practical issues faced by clinical nurses in general. Particularly, there is a notable gap in the literature regarding in-depth, targeted research on the sense of professional gain among pediatric nurses. This gap highlights the need for further investigation into the unique challenges and opportunities within pediatric nursing to inform targeted interventions and improve professional satisfaction. Given the importance of pediatric nursing in addressing the evolving healthcare needs, the purpose of this study is to gain an in-depth understanding of the current status and influencing factors of professional benefit perception among pediatric nurses. By identifying these factors, we aim to provide a scientific basis for nurse managers in clinical management and education. This study may also highlight the broader implications for policy-making, healthcare management, and nursing education, emphasizing the need for targeted interventions to enhance job satisfaction and promote the healthy growth of pediatric nurses. In this study, we utilized the Theory of Comfort developed by Katharine Kolcaba (17), which emphasizes the importance of comfort in nursing care across various dimensions, including physical, psychospiritual, environmental, and sociocultural contexts. This theory will guide our understanding of how pediatric nurses perceive professional benefits and the factors influencing these perceptions.

## Methods

### Ethical consideration

This study employed a cross-sectional survey design and was approved by the Ethics Committee of the Children's Hospital of Nanjing Medical University (approval number: 202410004-1). Written informed consent was obtained from all participants.

### Sample size consideration

According to the method for estimating sample size in multifactorial analysis (18), the sample size can be taken as 5–10 times the number of scale items (19, 20). The number of items in the scale for this survey is 29. To reduce errors and account for potential losses and invalid questionnaires during the collection process, the sample size was increased by 10%. Therefore, the minimum sample size for this study should be calculated as: (5×29×(1+10%)=159.5. Consequently, the survey should include at least 160 pediatric nurses.

### Participants

The survey selected pediatric nurses from our hospital from September 1, 2024, to November 26, 2024, as the study population. To ensure the rigor and relevance of our study, we carefully selected our sample of pediatric nurses based on specific inclusion and exclusion criteria. Participants were included if they held a valid professional qualification certificate, had a minimum of six months of clinical nursing experience in pediatrics, and provided informed consent to voluntarily participate in the study. Conversely, we excluded visiting nurses and nursing interns from other institutions who were temporarily assigned to our hospital, as well as those who were on external training or on sick/personal leave during the study period. Additionally, individuals who declined to participate were also excluded. These criteria were designed to ensure that our sample was both relevant and representative of the target population.

### Survey tool

To enhance the clarity and understanding of our research, we have defined several key factors that were included in our study, selected based on their potential influence on the professional benefit perception among pediatric nurses. The data collected for each nurse encompassed gender (categorized as male or female), age (recorded in years to reflect demographic characteristics), body mass index (BMI) calculated using the standard formula (weight in kilograms divided by height in meters squared) to assess general health status, years of experience in pediatric nursing (measured in years to capture the duration of clinical practice specifically in pediatric care), educational level (categorized based on the highest degree obtained, such as diploma, bachelor's degree, or master's degree), professional title (recorded to reflect current professional status, including titles such as registered nurse, senior nurse, or nurse manager), marital status (categorized as single, married, divorced, or widowed), working department (identified to reflect the specific unit or department within the hospital), childbearing status (recorded as having children or not having children to assess the impact of family responsibilities on professional perceptions), and average monthly income (measured in local currency to understand the financial aspect of job satisfaction). These factors were chosen to provide a comprehensive profile of the pediatric nurses included in our study and to identify potential correlates of professional benefit perception.

Besides, we used the previously reported and validated Nurses' Professional benefit perception Scale (21, 22) to evaluate the professional benefit perception of pediatric nurses. The Nurses' Professional benefit perception Scale was developed by Hu (23). This scale comprises 29 items across five dimensions: Personal Growth (6 items), Family and Friends' Recognition (7 items), Positive Nurse-Patient Relationships (6 items), Sense of Team Belonging (5 items), and Positive Occupational Perception (5 items). The scale utilizes a 5-point Likert scoring method, with options ranging from “Strongly Disagree” to “Strongly Agree,” assigned values from 1 to 5 respectively. All item scores are positively scored, with the total scale score ranging from 29 to 145, where a higher score indicates a stronger perceived sense of professional benefits. The overall Cronbach's *α* for the scale is 0.958, with dimension-specific Cronbach's α coefficients ranging from 0.821 to 0.893. The split-half reliability coefficients vary between 0.813 and 0.938, and the five dimensions account for 65.031% of the total variance. The Item-Content Validity Index (I-CVI) scores range from 0.83 to 1.00. The scale demonstrates satisfactory psychometric properties, meeting the standards for psychological measurement and exhibiting good reliability and validity (24, 25).

### Survey process

Before distributing the questionnaires, participants were provided with comprehensive information regarding the study's purpose and methodology, and their informed consent was obtained. It was clearly communicated that participation was voluntary, conducted anonymously, and that participants retained the right to refuse participation or withdraw at any time without consequence. Additionally, a pilot test was conducted with a small group of pediatric nurses to assess the clarity and reliability of the survey questions, and necessary refinements were made based on the feedback received. To ensure the integrity and accuracy of the data, all questionnaires were collected on-site and checked immediately upon completion. Investigators were available to provide clarifications for any survey items as needed. Furthermore, to address potential biases associated with on-site data collection, we implemented several measures. These included training data collectors to adhere to standardized procedures and reinforcing the voluntary nature of participation. All collected data were strictly used for the purposes of this research. These steps were taken to enhance the validity and reliability of our findings and to ensure that our methodology was robust and ethically sound.

### Statistical analysis

Data entry and analysis were conducted using SPSS 26.0 software. Qualitative data were presented as frequencies and percentages (%) and analyzed using the chi-square test (*χ*^2^ test). Quantitative data that conformed to a normal distribution were expressed as the mean ± standard deviation (x ± s) and analyzed using *t*-tests and one-way analysis of variance (ANOVA). Additionally, Pearson correlation and multiple liner regression analyses were employed to explore influencing factors of professional benefit perception of included pediatric nurses. Specifically, Pearson correlation was selected to assess the linear relationships between continuous variables, as it is a widely used and appropriate measure for this purpose. Multiple linear regression was chosen to identify and quantify the influence of multiple independent variables on a dependent variable, allowing us to control for potential confounders and better understand the underlying relationships in our data. The significance level for statistical tests was set at *α* = 0.05, with a *P*-value less than 0.05 considered to indicate statistically significant differences.

## Results

### Characteristics of pediatric nurses

A total of 366 pediatric nurses were included. As shown in Table 1, The surveyed pediatric nurses were predominantly female, with an average age of 31.19 ± 6.77 years. The average BMI of the nurses was 21.38 ± 2.04. The mean duration of pediatric nursing experience was 9.61 ± 3.85 years, and the majority of the nurses held a bachelor's degree.

**Table 1:** Characteristics of surveyed pediatric nurses (*n* = 366).

Characteristic	Cases	Professional benefit perception scores	t/F	*p*
Gender			1.216	0.113
Male	12 (3.28%)	106.65 ± 15.89		
Female	354 (96.72%)	108.41 ± 18.04		
Age			4.007	0.010
≤25	103 (28.14%)	99.24 ± 14.34		
26–35	144 (39.34%)	106.67 ± 16.55		
36–45	91 (24.86%)	110.95 ± 15.37		
≥46	28 (7.65%)	113.67 ± 14.60		
BMI(kg/m²)			3.124	0.097
<18.5	38 (10.38%)	107.95 ± 17.03		
18.5–24	277 (75.68%)	108.34 ± 16.17		
>24	51 (13.93%)	108.11 ± 17.64		
Years of experience in pediatric nursing			4.225	0.012
≤5	88 (24.04%)	101.62 ± 15.44		
6–10	136 (37.16%)	105.03 ± 16.69		
11–15	108 (29.51%)	109.77 ± 15.04		
≥16	34 (9.29%)	113.28 ± 14.71		
Educational level			2.055	0.019
Associate degree	130 (35.52%)	104.18 ± 16.77		
Bachelor's degree	232 (63.39%)	109.20 ± 15.42		
Master's degree	4 (1.09%)	109.36 ± 16.11		
Professional title			3.694	0.002
Junior nurse	115 (31.42%)	101.72 ± 16.08		
Nurse practitioner	142 (38.80%)	106.23 ± 17.05		
Charge nurse	105 (28.69%)	110.48 ± 16.39		
Deputy head nurse	4 (1.09%)	112.17 ± 13.90		
Marital status			3.007	0.001
Unmarried	121 (33.06%)	98.15 ± 14.80		
Married	245 (66.94%)	111.05 ± 16.43		
Working department			3.182	0.106
Department of internal medicine	90 (24.59%)	107.63 ± 15.16		
Department of surgery	86 (23.50%)	109.21 ± 17.43		
Department of outpatient	41 (11.20%)	108.55 ± 16.14		
Department of emergency	44 (12.02%)	107.48 ± 17.09		
Department of neonatology	49 (13.39%)	110.26 ± 16.33		
Intensive care unit	56 (15.30%)	106.12 ± 15.17		
Childbearing			1.277	0.114
Childless	152 (41.53%)	107.74 ± 16.43		
Has children	214 (58.47%)	108.79 ± 15.05		
Average monthly income (CNY)			3.252	0.005
<5,000	73 (19.95%)	101.06 ± 14.37		
5,000–10,000	214 (58.47%)	108.37 ± 15.75		
>10,000	79 (21.58%)	112.40 ± 16.08		

BMI, body mass index; CNY, China Yuan.

As shown in Table 2, the average score of professional benefit perception scores of included pediatric nurses was (108.26 ± 17.32), indicating the pediatric nurses have moderate level of professional benefit perception. Among the five dimensions, the highest scores were observed in the personal growth dimension; followed by the positive job perception dimension, while the lowest scores were found in the nurse-patient relationships dimension. As indicated in Table 1, there were statistical differences in the score of professional benefit perception among nurses with different age, years of experience in pediatric nursing, educational level, professional title, marital status and average monthly income (all *p* < 0.05). No statistical difference in the in the score of professional benefit perception among nurses with different gender, BMI, working department and childbearing were found (all *p* > 0.05).

**Table 2:** The professional benefit perception scores of included pediatric nurses.

Dimension	Number of items	Average score of each item	Total score
Personal growth	6	4.01 ± 1.04	23.89 ± 2.64
Recognition from family and friends	7	3.80 ± 1.12	26.42 ± 2.88
Good patient-nurse relationship	6	3.59 ± 1.16	22.60 ± 2.17
Sense of team belonging	5	3.64 ± 1.01	17.38 ± 2.29
Positive job perception	5	3.70 ± 1.14	17.66 ± 2.30
Total score	29	3.72 ± 1.05	108.26 ± 17.32

As shown in Table 3, Pearson correlation analysis revealed that age (*r* = 0.517), years of experience in pediatric nursing (*r* = 0.604), educational level (*r* = 0.564), professional title (*r* = 0.559), marital status (*r* = 0.623) and average monthly income (*r* = 0.647) were correlated with the professional benefit perception scores of included pediatric nurses (all *p* < 0.05).

**Table 3:** Pearson correlation analysis on the relationship of characteristics and professional benefit perception scores of included pediatric nurses.

Characteristic	*r*	*p*
Gender	0.109	0.233
Age	0.517	0.040
BMI	0.116	0.104
Years of experience in pediatric nursing	0.604	0.028
Educational level	0.564	0.015
Professional title	0.559	0.004
Marital status	0.623	0.013
Working department	0.176	0.102
Childbearing	0.160	0.241
Average monthly income	0.647	0.001

BMI, body mass index.

As indicated in Table 4, Multiple linear regression analysis revealed that age, years of experience in pediatric nursing, educational level, professional title, marital status and average monthly income were the influencing factors of professional benefit perception scores of pediatric nurses. The six factors accounted for 57.20% of the total variance in the professional benefit perception of pediatric nurses (all *p* < 0.05).

**Table 4:** Multiple linear regression analysis on the influencing factors of professional benefit perception scores of pediatric nurses.

Variables	Regression coefficient	Standard error	Standardized coefficient	*t*	*p*
Constant	50.558	2.686	–	21.205	<0.001
Age	3.416	0.792	0.432	3.117	0.027
Years of experience in pediatric nursing	2.956	0.803	0.328	3.890	0.013
Educational level	4.009	0.635	0.473	4.667	0.002
Professional title	6.765	1.681	0.309	3.922	0.025
Marital status	4.004	0.817	0.794	4.008	0.039
Average monthly income	6.175	1.188	0.303	5.368	0.001

*R*^2^ = 0.598, adjusted *R*^2^ = 0.572, *F* = 58.046, *p* < 0.001.

## Discussion

The results of this study indicate that the professional benefit perception among pediatric nurses are generally at a moderate level, slightly lower than the findings of previous related research (26–30), which may be attributed to differences in the study population and location. In this study, the dimension of personal growth scored the highest, likely due to the emphasis hospital administrators place on the comprehensive development of nurses' qualities, thereby fostering personal growth among nurses. Concurrently, the conditions of pediatric patients are often critical, necessitating that nurses address the needs of both the patients and their families (31). This situation compels pediatric nurses to accelerate their pace of self-improvement and continuously enhance their professional competencies to better perform their daily nursing duties (32, 33). On the other hand, the nurse-patient relationships dimension scored the lowest, consistent with the findings in existing literature (34, 35). This phenomenon may be related to the unique work environment of pediatrics.

Given the particularities of pediatric patients, family members often have higher expectations and demands, and the complexity of nursing procedures can lead to a higher likelihood of medical disputes (36, 37). Therefore, nurse managers should implement targeted measures to enhance the professional benefit perception and benefits for pediatric nurses, thereby optimizing nurse-patient relationships and improving the overall quality of nursing care.

Enhancing the perception of professional benefits among pediatric nurses can have a significant positive impact on both patient care and organizational outcomes. For instance, improved professional benefits perception can lead to higher job satisfaction and motivation among nurses, which in turn can enhance the quality of patient care (11, 12). A motivated nursing workforce is more likely to engage in patient-centered care practices, leading to better patient outcomes, increased patient satisfaction, and reduced healthcare costs (38). Additionally, when nurses feel valued and supported, they are less likely to experience burnout, which is critical for maintaining a stable and competent nursing workforce. From an organizational perspective, addressing the factors that influence professional benefits perception can lead to improved staff retention, better crisis management, and a stronger organizational reputation (39). Implementing strategies such as continuous professional development, clear career advancement pathways, and supportive work environments not only benefits individual nurses but also contributes to the overall efficiency and effectiveness of healthcare organizations (40).

There is a positive correlation between the overall scores and individual dimensions of professional benefit perception among pediatric nurses and their age, indicating that as age increases, so does the sense of professional benefits. This trend is likely associated with the accumulation of work experience and life experiences that come with age. Nurses with more years of service typically possess richer work experience and life wisdom, enabling them to face various challenges at work with greater confidence and composure (27–30, 41). Furthermore, as the duration of service grows, these nurses often accumulate higher prestige within their departments and may hold middle to senior management positions, which further enhances their sense of belonging and identification with their work (42).

On both personal and professional levels, the accumulation of work experience leads to significant enhancements in nurses' knowledge and professional skills. This progression fosters a deeper appreciation and recognition of their work. Moreover, it equips them with greater proficiency in navigating career development and personal growth within their field (27–30). As work experience expands, the positive feedback and support nurses receive in terms of personal performance, social recognition, and job acknowledgment also increase continuously (43, 44). This not only elevates their personal prestige within their workplace but also strengthens their sense of belonging. Consequently, over time, the scores of professional benefit perception among nurses tend to rise, reflecting their positive attitude towards their career and a profound understanding of the value of their work (45).

There is a significant correlation between the level of professional benefit perception and the educational level of pediatric nurses, with the sense of professional benefits generally increasing as educational levels rise. Nurses with higher education often better utilize their extensive knowledge and skills, as well as unique ways of thinking, to promote personal career growth. These nurses tend to receive higher recognition in their work and society, and correspondingly better compensation, thus making them more likely to be valued (46). Due to the attention and training provided by their institutions and leaders, they can quickly enhance their sense of personal value within the team and successfully establish a sense of belonging. However, some scholars (27–30, 47) hold a different view, suggesting that nurses with higher education may have a lower sense of professional benefits. This may be related to the higher self-expectations of nurses with higher education. Therefore, nurse managers should establish training systems at different levels according to the needs of nurses with different educational backgrounds, providing the maximum space for development to help them achieve personal value.

Furthermore, the higher the professional title of pediatric nurses, the higher their level of professional benefit perception. Nurses with higher professional titles usually have longer working hours and richer clinical nursing experience. Their career planning, life values, and sense of team belonging are more defined, thus enabling them to perceive a greater sense of professional benefits (43, 44, 48). This indicates that as professional experience accumulates and personal abilities improve, nurses can better experience the intrinsic satisfaction and external recognition that work brings.

Married pediatric nurses exhibit a higher sense of professional benefits compared to their unmarried counterparts, a phenomenon that can be explained from various perspectives. Firstly, unmarried nurses often belong to the younger group within their departments, being at the initial stage of their career where they need to devote substantial time to learning and training. The lack of work experience subjects them to relatively higher work pressures, which directly affects their level of professional benefit perception (49). Furthermore, when nurses divert part of their attention to family life, they may adopt a more indifferent attitude towards negative work situations, a mindset that helps reduce work pressure and, to some extent, enhances their sense of professional benefits and work responsibility (50). Family support not only provides emotional comfort for nurses but also aids in better psychological adjustment and career development along their professional journey (51). Therefore, medical institutions, while focusing on the career development of nurses, should also recognize the positive impact of family support in enhancing the sense of professional benefits among nurses.

With the increase in monthly income, the overall sense of professional benefits and its various dimensions among nurses show an upward trend. The level of salary, as an important indicator of material security, not only enhances nurses' recognition and satisfaction with their work but also reflects the principle that the efforts nurses put into their work should be proportional to the rewards they receive, which is a key factor in reducing job burnout (52). A higher salary level typically implies more opportunities for personal growth and development. Furthermore, the salary level symbolizes an individual's social status to a certain extent (53). Higher incomes often lead to greater recognition from family and society, which further clarifies the positive perception nurses have of their profession and strengthens their proactive attitude and sense of responsibility towards their work (54). Consequently, as income increases, nurses may gain more positive experiences in career development, social recognition, and work engagement, thereby enhancing their overall sense of professional benefits (55). This positive cyclical effect emphasizes the significant role of a fair compensation system in motivating nurses, promoting their career development, and improving job satisfaction.

Understanding the key determinants of professional benefit perception among pediatric nurses—such as years of experience, educational level, and work environment—provides a critical foundation for developing targeted strategies to enhance job satisfaction and professional benefits. For example, identifying that nurses with more experience may perceive higher professional benefits could inform the design of mentorship programs, pairing experienced nurses with less experienced colleagues to foster professional growth and satisfaction. Similarly, recognizing the impact of educational level suggests that hospitals could invest in continuous education and training programs to support nurses in advancing their qualifications. Insights into the work environment highlight the importance of creating supportive workplace policies, such as flexible scheduling, adequate staffing levels, and recognition programs, which can improve nurses' overall job satisfaction and well-being. Combined with initiatives to improve the overall work environment, such as enhancing communication channels and addressing workplace stressors, these strategies can collectively contribute to a more motivated and satisfied pediatric nursing workforce.

There is significant room for improvement in the professional benefit perception among pediatric nurses. Previous study has proposed that adopting the social embeddedness model of job flourishing as a management framework can provide administrators with a systematic set of organizational management strategies (56). These strategies include empowering nurses, creating a positive organizational climate, strengthening solidarity among colleagues, and enhancing connectivity with leaders, which can help pediatric nurses perceive more professional benefits in their work processes. Therefore, exploring and implementing various measures to enhance nurses' positive occupational perception should be a key focus for government functional departments, medical institutions, and administrators (57).

While this study possesses exploratory value, it is not without limitations that require attention. Firstly, the survey was confined to pediatric nurses from a single tertiary hospital in China, with a relatively small sample size and a limited sampling scope, which may restrict the broad applicability of the study's findings and potentially introduce biases in certain conclusions. We acknowledge that while our sample includes pediatric nurses with diverse experience levels, future studies may benefit from exploring geographical diversity to better capture the broader population of pediatric nurses. Secondly, while self-reported data are an essential component of our study, we acknowledge the inherent biases that may arise. To mitigate the self-reported survey bias, we implemented several measures during data collection, such as emphasizing the anonymity and voluntary nature of participation, providing clear instructions, and pre-testing the survey to ensure clarity and reliability. Thirdly, it is important to acknowledge that the assessment of protective factors through psychotherapy and counseling interventions is a significant consideration, especially given the substantial work burden often experienced by health professionals. However, our survey did not include specific questions to evaluate the use of psychotherapy or counseling interventions as protective factors. This omission represents a limitation of our study. Future research should consider incorporating questions related to the utilization of psychotherapy and counseling interventions to provide a more comprehensive understanding of how health professionals manage their well-being.

## Conclusions

In summary, our study shows that pediatric nurses' perception of professional benefits is currently moderate, indicating significant potential for improvement. Key factors influencing this perception include age, years of pediatric nursing experience, educational level, professional title, marital status, and average monthly income. These findings highlight the need for targeted interventions to enhance professional benefits. By addressing these factors through strategic interventions, nursing managers can enhance pediatric nurses' professional benefits, strengthen their professional identity, and improve the quality of care delivered to pediatric patients. Future research should explore the effectiveness of these interventions in diverse settings and populations.

## Data Availability

The original contributions presented in the study are included in the article/Supplementary Material, further inquiries can be directed to the corresponding authors.

## References

[1] Liu C Zhang Z Zhang Y. Research progress on the concept and framework of integrated health management service models for children at home and abroad. Chin Gen Pract Med. (2024) 27(28):3574–80.

[2] Geng Y Zhuo L Zhang R Zhao H Hou X Chen H The impact of China’s universal two-child policy on total, preterm, and multiple births: a nationwide interrupted time-series analysis. BMC Public Health. (2024) 24(1):236. 10.1186/s12889-023-17620-5 38243163 PMC10799358

[3] Wang J Zhang L. Public policies for fertility recovery: constraints, incentives and support. Popul Dev. (2024) 30(1):2–12.

[4] Ji W Huang L Xu A. Research on the shortage of pediatricians in Jiangsu from a comprehensive perspective of supply-demand. Chin Gen Pract Med. (2024) 27(7):829–33.

[5] Jeon BY Yun SJ Kim HY. Factors influencing job stress in pediatric nurses during the pandemic period: focusing on fatigue, pediatric nurse-parent partnership. J Spec Pediatr Nurs. (2024) 29(4):e12437. 10.1111/jspn.12437 39183593

[6] Ozsavran M Kurt A Ayyildiz TK Gul Z. “A life slips through our fingers” experiences of nurses working in pediatric intensive care units about children’s death: a qualitative study. Omega (Westport). (2024) 10:302228231225885. 10.1177/00302228231225885 38166543

[7] Dong SS Wang K Zhang KQ Wang XH Wang JH Turdi S Decision fatigue experience of front-line nurses in the context of public health emergency: an interpretative phenomenological analysis. BMC Nurs. (2024) 23(1):553. 10.1186/s12912-024-02163-w 39135083 PMC11321180

[8] Shao H Gan H Wei T. Investigation on the current situation of burnout and resignation intention of pediatric nurses in Chongzhou. Ind Hyg Occup Dis. (2022) 32(4):48–52.

[9] Shen Y Zhou W Xiao Y. Construction of a prediction model for severe burnout in clinical nurses. J Nurs Manag. (2023) 23(10):797–802.

[10] Xi L Song J Yang S. Correlation analysis of role stress, psychological capital and burnout among 216 pediatric nurses in 5 tertiary A hospitals in Chengdu. Occup Health. (2022) 36(13):38–42.

[11] Zhang H Hoe VCW Wong LP. The association between burnout, perceived organizational support, and professional benefit perception among nurses in China. Heliyon. (2024) 10(20):e39371. 10.1016/j.heliyon.2024.e39371 39498039 PMC11532839

[12] Zhang Y Meng X Zhou L. The impact of job stress on professional benefit perception among Chinese nurses caring for patients with gynecological cancer: mediating effects of perceived social support and self-efficacy. Front Psychol. (2024) 15:1344185. 10.3389/fpsyg.2024.1344185 38633878 PMC11021783

[13] Wan J Zhou W Qin M Zhou H. The effect of professional benefit perception on health professionals’ job engagement: the role of psychological availability and future professional benefit perception. BMC Health Serv Res. (2024) 24(1):227. 10.1186/s12913-024-10684-y 38383405 PMC10882821

[14] Lin Y Jiang C Pan Y Xu Z. The impact of mindfulness on nurses’ professional benefit perception: the mediating roles of workplace spirituality and work-life balance. Front Psychol. (2024) 15:1346326. 10.3389/fpsyg.2024.1346326 38476383 PMC10929680

[15] Chen Y Liu L Wang Y. Correlation between role stress and career benefit in pediatric nurses. Contemp Nurs. (2024) 31(1):164–7.

[16] Deng KQ Chen XY Yuan XM Ren YR Luo ZM Li GY The effect of nurses’ perceived stress on their work engagement and perceived professional benefit during the routine management of the COVID-19 pandemic. Work. (2024) 78(4):873–81. 10.3233/WOR-220498 38160380

[17] Kolcaba K Tilton C Drouin C. Comfort theory: a unifying framework to enhance the practice environment. J Nurs Adm. (2006) 36(11):538–44. 10.1097/00005110-200611000-00010 17099440

[18] Yuan J Li X. Comparative study on sample size calculation methods. Stat Decis Mak. (2013) 22(1):4–8.

[19] Fang J Wu W Jin L. Investigation on the current situation of occupational stress and burnout among pediatric nurses. Chin J Mod Nurs. (2024) 30(27):3755–8.

[20] Guan P Chen J Sun Y. Investigation on knowledge, attitude and practice on prevention of venous catheter-related bloodstream infections in nurses ‘centers in children’s specialized hospitals. Chin Health Qual Manag. (2024) 31(6):50–6.

[21] Hu J Liu X. Development and reliability and validity of nurses ‘occupational benefit questionnaire. PLA J Nurs. (2013) 30(22):5–7.

[22] Hu J Liu X. Qualitative study on the conceptual framework of “nurses ’sense of professional benefit”. J Nurs Educ. (2014) 29(8):4–9.

[23] Hu J. Research on the Conceptual Framework and Evaluation Tools of Nurses ‘Professional Benefit. Shanghai: Second Military Medical University (2013).

[24] Kang J Zhang Q Zhang W. The current situation of nurses’ sense of professional benefit in a third-level A children’s specialized hospital in Jiangxi province. Gen Pract Nurs. (2023) 21(4):554–8.

[25] Zheng S Wang N Jing W. Correlation between professional benefits and core competencies of pediatric nurses. Evid Based Nurs. (2023) 9(12):2231–4.

[26] Du Y Zhang Y Lu X. Survey on the subjective social status and influencing factors of nurses in 19 tertiary hospitals in Shanghai. J Nurs. (2023) 38(14):54–9.

[27] Wang J Zhang G Li G. Analysis of the current situation and influencing factors of professional benefits among ICU specialist nurses. Nurs Pract Res. (2023) 20(6):841–5.

[28] Wang M Lu C Wang L. A study on the current situation and influencing factors of nurses’ sense of professional benefit in top three hospitals in Wuhan. Chin J Soc Med. (2023) 40(6):709–13.

[29] Wang M Wang L Lu C. Nurses’ sense of organizational support, self-esteem and professional benefit perception: a mediating model. Nurs Open. (2023) 10(4):2098–106. 10.1002/nop2.1457 36490363 PMC10006623

[30] Wang X Chen F Dai P Lin X Qi L. Professional benefit perception and associated factors among nurses during the COVID-19 pandemic: a cross-sectional study. Nurs Open. (2023) 10(3):1461–70. 10.1002/nop2.1396 36176012 PMC9538648

[31] Benini F Mercante A Di Nunzio S Papa S, PalliPed Working G. The specialized pediatric palliative care service in Italy: how is it working? Results of the nationwide PalliPed study. Ital J Pediatr. (2024) 50(1):55. 10.1186/s13052-024-01604-1 38504292 PMC10953081

[32] Blixt C Johansson E Forsner M Angelhoff C. Compassion fatigue and compassion satisfaction in pediatric and neonatal care nurses during the COVID-19 pandemic in Sweden. J Pediatr Nurs. (2023) 73:e646–51. 10.1016/j.pedn.2023.11.013 37977972

[33] Judith Roach E Al Omari O Elizabeth John S Francis F Arulappan J Shakman L Challenges experienced by nurses in providing pediatric palliative care: an interpretive phenomenological analysis. J Palliat Care. (2023) 38(3):355–63. 10.1177/08258597231173313 37143338

[34] Ding Q Bi L Liu N. Analysis of current situation and influencing factors of occupational burnout among nurses in pediatric wards. Chin Hosp Stat. (2023) 30(5):345–52.

[35] Hao J Zhang Q Wu X. Empathy status of pediatric nurses and its relationship with burnout and quality of professional life. Nurs Pract Res. (2023) 20(9):1294–8.

[36] Beardsley E Patton L Reynolds C Williams A Hernandez E Chen P Evidence-based approach to mitigate cumulative stress in pediatric nurses through the development of respite rooms. Worldviews Evid Based Nurs. (2024) 21(1):96–103. 10.1111/wvn.12702 38189600

[37] Yehene E Asherman A Goldzweig G Simana H Brezner A. Secondary traumatic stress among pediatric nurses: relationship to peer-organizational support and emotional labor strategies. J Pediatr Nurs. (2024) 74:92–100. 10.1016/j.pedn.2023.11.019 38029691

[38] Ortiz MR. Best practices in patient-centered care: nursing theory reflections. Nurs Sci Q. (2021) 34(3):322–7. 10.1177/08943184211010432 34212801

[39] Nashwan AJ Cabrega JA Othman MI Khedr MA Osman YM El-Ashry AM The evolving role of nursing informatics in the era of artificial intelligence. Int Nurs Rev. (2025) 72(1):e13084. 10.1111/inr.13084 39794874 PMC11723855

[40] Tiittanen H Heikkila J Baigozhina Z. Development of management structures for future nursing services in the Republic of Kazakhstan requires change of organizational culture. J Nurs Manag. (2021) 29(8):2565–72. 10.1111/jonm.13416 34252232 PMC9291935

[41] Zhou LH Stenmarker M Henricson M Mengi XH Zhang YX Hong JF Professional benefit perception and their associated factors among Chinese registered nurses caring for women diagnosed with gynecological cancer. Eur J Oncol Nurs. (2023) 66:102345. 10.1016/j.ejon.2023.102345 37689047

[42] Sun C Jiang H Yao Q Wang X Wen X Liu H. Latent profile analysis of nurses’ professional benefit perception in China: a cross-sectional study. BMJ Open. (2023) 13(11):e078051. 10.1136/bmjopen-2023-078051 37918934 PMC10626806

[43] Li L Feng Z Zhu M Yang J Yang L. The mediating effect of personality on mental workload and professional benefit perception of nurses in east China. BMC Nurs. (2023) 22(1):440. 10.1186/s12912-023-01603-3 37993932 PMC10664375

[44] Li Q Zheng D Rui X. Correlation between clinical nurses ‘training load and career benefit. Gen Pract Nurs. (2023) 21(11):1570–3.

[45] Friedrich AB Kohlberg EM Malone JR. Perceived benefits of ethics consultation differ by profession: a qualitative survey study. AJOB Empir Bioeth. (2023) 14(1):50–4. 10.1080/23294515.2022.2093423 35856904

[46] Alamri H. Instructors’ self-efficacy, perceived benefits, and challenges in transitioning to online learning. Educ Inf Technol (Dordr). (2023) 6:1–36. 10.1007/s10639-023-11677-w 37361801 PMC10126569

[47] Su W Xizhang P Chen Y. Current situation of professional identity among nurses in nephrology department in Wuzhou and its correlation with work pressure and professional benefits. Occup Health. (2023) 39(7):928–31.

[48] Wu X Zhu C Xu Z. The mediating role of psychological capital in rehabilitation specialist nurses in clinical leadership and career benefits. Evid Based Nurs. (2024) 39(14):10–3.

[49] Sexton JR Truog AW Kelly-Weeder S Loftin C. The effects of moral distress on resilience in pediatric emergency department nurses. J Emerg Nurs. (2024) 50(5):626–34. 10.1016/j.jen.2023.10.006 38300203

[50] Deschenes S Scott SD Kunyk D. Mitigating moral distress: pediatric critical care nurses’ recommendations. HEC Forum. (2024) 36(3):341–61. 10.1007/s10730-023-09506-1 37140806 PMC10158695

[51] Roney LN Rankin G Robertson B Budd T Zaino K Sylvestre V Caring through crisis: the professional quality of pediatric nurses during the COVID-19 pandemic. J Pediatr Nurs. (2024) 12:14–20. 10.1016/j.pedn.2024.07.019 39129084

[52] Geven BM Maaskant JM van Woensel JBM Verbruggen S Ista E. Barriers and perceived benefits of early mobilisation programmes in Dutch paediatric intensive care units. Nurs Crit Care. (2023) 28(4):519–25. 10.1111/nicc.12841 36151585

[53] Marzanek F Nair K Ziesmann A Paramalingam A Pirrie M Angeles R Perceived value and benefits of the community paramedicine at clinic (CP@clinic) programme: a descriptive qualitative study. BMJ Open. (2023) 13(11):e076066. 10.1136/bmjopen-2023-076066 37989376 PMC10668171

[54] Antonacci G Benevento E Bonavitacola S Cannavacciuolo L Foglia E Fusi G Healthcare professional and manager perceptions on drivers, benefits, and challenges of telemedicine: results from a cross-sectional survey in the Italian NHS. BMC Health Serv Res. (2023) 23(1):1115. 10.1186/s12913-023-10100-x 37853448 PMC10585875

[55] Peng C Chen Y Zeng T Wu M Yuan M Zhang K. Relationship between perceived organizational support and professional values of nurses: mediating effect of emotional labor. BMC Nurs. (2022) 21(1):142. 10.1186/s12912-022-00927-w 35668396 PMC9169319

[56] Nguyen LH Nguyen LTK Nguyen TT Trong Dam VA Vu TMT Nguyen H Practices, perceived benefits, and barriers among medical students and health care professionals regarding the adoption of eHealth in clinical settings: cross-sectional survey study. JMIR Med Educ. (2022) 8(3):e34905. 10.2196/34905 36098992 PMC9516362

[57] Zhao Y Zhang L Hua L. A bibliometric analysis of research on nurses’ sense of professional benefit in China. Hainan Med. (2023) 34(19):2855–9.

